# ‘Love at first sight’: The effect of personality and colouration patterns in the reproductive success of zebrafish (*Danio rerio*)

**DOI:** 10.1371/journal.pone.0203320

**Published:** 2018-09-19

**Authors:** Reynaldo Vargas, Simon Mackenzie, Sonia Rey

**Affiliations:** 1 Departamento de Biología Marina y Limnología, Extensión Universitaria de Aguadulce, Universidad de Panamá, Aguadulce, Coclé, República de Panamá; 2 Institute of Aquaculture, University of Stirling, Stirling, Scotland, United Kingdom; Oregon State University, UNITED STATES

## Abstract

Individual differences in animal personality and external appearance such as colouration patterns have both been extensively studied separately. A significant body of research has explored many of pertinent ecological and biological aspects that can be affected by them and their impact upon fitness. Currently little is known about how both factors interact and their effect on reproductive success. In this study, we evaluated two major parameters contributing to the fitness of the species: reproduction and offspring survival. We selected two different phenotypes of the zebrafish (*Danio rerio*) selected by their colouration patterns: phenotype 1) named Wild type, UAB line (WT-UAB) with a homogeneous colouration pattern (clear and defined lateral stripes) and phenotype 2) Wild type indefinite (WT-I) had a heterogeneous colouration pattern and different degrees of lateral stripe definition. All animals were also screened for personality. We then compared their reproductive success (spawning rate) and offspring survival at different stages, from egg to larvae, and for 2 successive generations (parental generation was G0; First and second generations were G1 and G2 respectively). Our results show that personality traits were the main source of variability between the fitness components measured: both personalities had similar total numbers of eggs spawned but proactive animals, for both colour phenotypes, had higher reproductive success. This was reflected in a higher percentage of spawning viability at 1day post fertilization (dpf), higher total survival and growth rates at larval stages. Proactive phenotypes from WT-UAB population had a higher overall fitness in comparison to the other phenotypes studied. Our findings imply that fitness of this species when kept under similar husbandry conditions is significantly influenced by parental personality and not by their external appearance. Under these conditions the reproductive success is enhanced. The implications of this study are important for zebrafish breeding and husbandry in captivity and are relevant toward understanding the underlying drivers of trait selection in natural environments.

## Introduction

Individual variation is the raw material for evolution and so it is important to understand how some traits are inherited and maintained across generations. Differences in behavioural phenotypes and external coloration both have impact on the fitness of the species however both have been separately studied. Previous studies relating to animal behavioural characteristics (more proactive parental care, courtship intensity, dominance and agonistic behaviours) and coloration to reproductive success in birds and fish [1–4] suggest a linkage between these traits. It has been hypothesized that personality together with other parameters like sexual and ecological isolation could be a driver of reproductive isolation leading to speciation [5]. In this study we link coloration patterns with personality and examine their interactive effects on reproductive success.

Individual differences in behaviour among individuals in a population are well documented [6–8]. When individual variation is consistent and repeatable over time and across contexts we define this variation as animal personalities [9,10]. Several terms are used to categorize the extremes of variation in behavioural responses [7,11,12]; in this study, we used the proactive (P)-reactive (R) axis [13]. P individuals are characterized by consistently being bolder and taking more risks than their R counterparts; other behaviours have been correlated with this proactive personality such as establishing routines, success in stable and resource-rich environments, early reproduction and low basal cortisol levels amongst others [14–19]. This individual variation has been described in several species of fish, for example [20] rainbow trout (*Oncorhynchus mykiss)*; [21] sticklebacks (*Gasterosteus aculeatus)*; [22] African catfish (*Clarias gariepinus)*; [23] Senegalese sole (*Solea senegalensis)*; [24] carp (*Cyprinus carpio)* and [13] zebrafish (*Danio rerio*). Personality traits used by the individual to cope with various environmental changes have direct implications on their physical performance or "fitness" [25,26]. Direct relationships between behaviour and fitness have been shown in some species such as the bighorn sheep (*Ovis canadensis*) where bolder individuals had better survival rates in times of high predation pressure [27]. In birds, studies found fast scan positively related to aggression and competition, and their consequences for fitness were reflected in a higher annual adult survival [28,29]. In fish, there is less literature examining the relationship between personality and fitness of the species [30–33]. However, there is evidence that personality traits such as boldness, activity or aggressiveness are related to reproductive success including offspring survival. It has been suggested that P individuals show greater reproductive success and better growth rates than R individuals in the wild under favourable environmental conditions and stable environments[34,35]. This has also been suggested under standard laboratory conditions [32,36] using zebrafish and guppies (*Poecilia reticulata*) as model species.

Individual differences in colouration patterns in animals are associated with multiple signals, most of them related to dominance, sexual selection, individual quality and health [37]. Colouration in fish is often associated with male-female differences [38,39] mostly for reproduction purposes (for example in lumpfish, *Cyclopterus lumpus* or Nile tilapia, *Oreochromis niloticus*). The zebrafish is a small cyprinid native to South Asia, widely distributed across India, Bangladesh, Nepal, Myanmar and Pakistan [40,41]. Zebrafish (*Danio rerio*) are not clearly dimorphic but display bright colouration patterns in the form of several lateral blue and golden stripes crossing their body cranio-caudally (this is the origin of the name, resembling the stripes of a zebra) that can be slightly different for mature males and females. The reason for this colouration pattern is unknown but could be a defensive mechanism, contributing to predator confusion [42,43], as they are a highly social shoaling fish in the wild [40,41]. Individuals with defined stripes could also indicate a higher social rank order (more dominant)[44] and therefore better stripe definition may be a positive selection trait within the population [45]. Defined and brighter coloration traits may also be indicative of better health status (related to the MHC: Major Histocompatibility Complex) and so positively selected within the population [46]. Similar stripe patterns reinforce social aggregations with Danios being able to discriminate conspecifics from heterospecifics [47] and promote assortative mating [48].

Zebrafish reproduction in nature is associated with the monsoon cycle however for fish bred under laboratory conditions reproduction is all year round [49,50]. Studies related to certain reproductive parameters vary within this species [51]. However, variables such as female fecundity have been linked to spawning intervals, sex, age and size [52–54]. Despite the importance of zebrafish in biological research little is known about the effect of external colouration and behavioural phenotypes on reproductive success and larval growth. The main objective of this study was to evaluate the effect of personality on male and female reproductive success for two selected colour phenotypes. We further investigated whether the colouration and personality of the parents had a role in larval growth and offspring survival. Based on the results from previous studies our hypothesis was that proactive zebrafish with clear, better defined colouration patterns would have more and better offspring therefore higher reproductive success leads to higher fitness. To test this hypothesis, we selected zebrafish according to external colouration patters (lateral stripes) and to their personality. We crossed animals accordingly and measured several reproductive parameters from spawning to embryo and up to larval stages to evaluate reproductive fitness and larval growth. We also measured differences in female guarding behaviour during mating between proactive and reactive males. To our knowledge this is the first study linking reproductive success with colour and behavioural phenotypes in the model organism, zebrafish.

## Material and methods

### Broodstock lines selection and housing conditions

**Fig 1:**
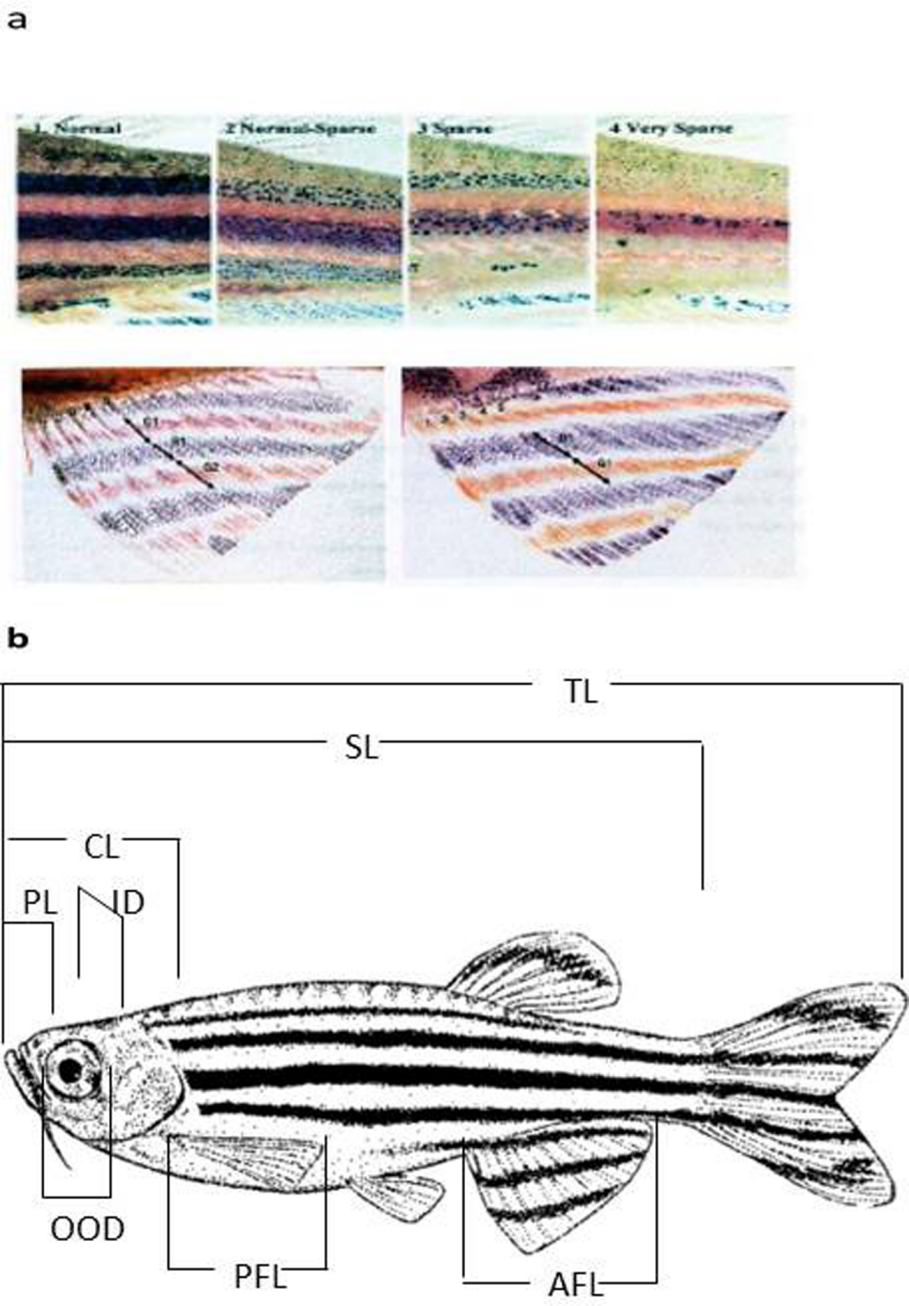
Phenotypic selection criteria for colouration patterns and morphometric measurements taken from zebrafish. (a) Colouration patterns used to select zebrafish colour phenotypes (WT-UAB and WT-I). Following the different sequential criteria from Table 1; (b) Morphometric measurements taken for all zebrafish adults in the G0 population studied (TL: Total Length, SL: Standard Length, CL: Cephalic Length, PL: Preocular Length, AFL: Anal fin Length, PFL: Pectoral fin length, OOD: Ocular Orbital Diameter, ID: inter-orbital distance). Reproduced from Pritchard [56] with permission from the author and University of Leeds.

**Fig 2:**
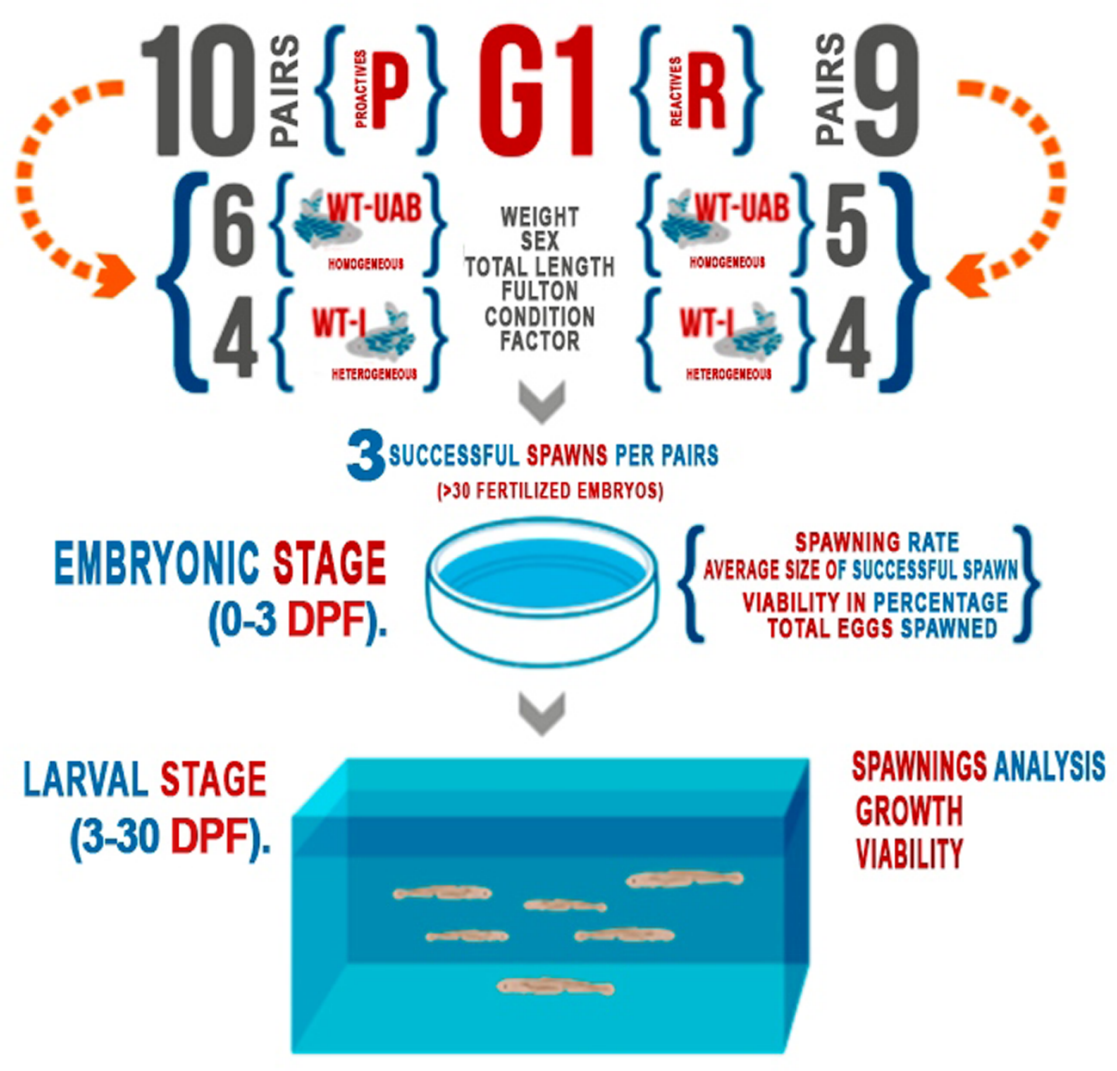
Experimental set-up and flow. Generation 1 (G1) crossing pairs divided into colour and personality phenotypes and the morphometric measurements and reproductive variables taken at each developmental stage.

The broodstock, Wild Type (short fin) unspecified zebrafish (*Danio rerio*) line (WT as defined by ZFIN.org) used to develop this experiment were adults between 10 and 12 months old, sourced from our selection program for personality lines in [55] bred and raised at the zebrafish facility of the Institute of Biotechnology and Biomedicine at the Autonomous University of Barcelona or UAB (Spain). Parental generation of fish (G0) from our stock population were first selected for colouration patterns: Wild type, UAB (University Autonomous of Barcelona) line or WT-UAB were selected for homogeneous colouration patterns as described in wild-type populations (as described in [56]) and the remaining fish with heterogeneous colouration patterns were classified as wild type indefinite or WT-I. Fish were anesthetised, placed individually on a petri dish with filtered water from the holding tanks at 28°C and observed under a binocular lens. The operational variables to select for colouration patterns were defined according to 1) Uniformity of colour at both sides of the body: similar number of stripes and stripe pattern. From four to five dark stripes (blue) and 4 clear stripes (golden) in both lateral sides of the body. 2) Colour lateral stripes (blue and golden) well defined at both sides of the body: uniformity in stripe pattern regarding orientation, thickness and colour integrity in both lateral sides of the body. The integrity is considered when the stripe keeps the same thickness and density of colour in all their extension along the body. 3) Right insertion of the body lateral stripes into the caudal fin: the stripes coming from both sides of the body follow the same trajectory in the caudal fin. 4) Straight and well-defined anal colour stripes: stripes keep their orientation, thickness and integrity (as defined above) in both sides of the anal fin (see Fig 1A and Table 1 for phenotypic characteristics of each colouration pattern and sequential classification criteria and Fig 2 for experimental set-up). The observation sequence goes from more general to more specific colour phenotypic characteristics. Animals with a score of 4 (meaning they were positive for all the characteristics mentioned above) were classified as homogeneous and went into the WT-UAB group. Animals that scored 3 or less went into the WT-I group independently of which characteristic scored negatively.

**Table 1:** Sequential observation order and criteria used to classify between both colour phenotypes within the base population of WT zebrafish at our facility. The observation sequence goes from more general to more specific colour phenotypic characteristics.

**PHENOTYPIC CHARACTERISTICS**	**CRITERIA**	
	WT-UAB	**WT-I**
**1. Uniformity of colour at both sides of the body**	*+*	*_*
**2. Colour lateral stripes (blue and golden) well defined at both sides of the body**	*+*	*_*
**3. Right insertion of the body lateral stripes into the caudal fin.**	*+*	*_*
**4. Straight and well defined anal colour stripes.**	*+*	*_*

All parental fish (G0) from each phenotypic colour line (WT-UAB and WT-I) were screened ‘*a posteriori’* for personality using 4 independent personality tests (risk-taking in groups, mirror stimulation test, feeding, novel environment test) previously validated in our group for this species [13]. From G0 we crossed animals according to colour and behavioural phenotype and then re-screened their progeny (G1) for personality (by a risk taking in groups test). Once adult fish were screened for colouration patterns detailed morphometric measurements were carried out (see Fig 1B; and found explained in [13,55].

Fish were held in pairs only for reproductive purposes. Each fish in each pair was individually held in methacrylate tanks of 1.4 L (Aquaneering**®** ZT140; Dimensions (L X W X H): 26.5 X 5.4 X 14.0 cm) arranged on shelves in a room within a recirculating System (RAS) under a controlled environment. The feeding pattern was modified from the 15th day prior to the start of the reproduction and maintained during the course of the experiment, adding a third supply of food at noon, following the recommendations of the food supplier (ZfBiolabs®). Additionally, to improve the quality of the spawning, twice a week throughout the course of the experiment, the broodstock received a supplement of brine shrimp (*Artemia sp*., Ocean Nutrition ®, Esse, Belgium).

The water supply was pre-filtered tap water (CTO ® cartridge filter, Water Quality Association, Lisle, IL, USA). Ambient and water temperature was maintained at 28 ±1.00°C, oxygen concentrations were maintained at 6.67±0.26mg/L, both measurements were performed daily with an oximeter (OxyGuard ®, Handy Polaris OxyGuard International, Denmark). Additionally, weekly tests of Ammonia, Nitrite, Nitrate and pH (Sera® Test Kit) were performed. The photoperiod was set up at 14:10 h day: night (06:00–20:00 hours) for reproduction purposes.

All protocols and animal experiments were approved by the Institutional Animal Care committee (OH-CEEA-UAB: Organ Habilitat, Comitè Ètic d'Experimentació Animal (CEEA) de la Universitat Autònoma de Barcelona (UAB) http://www.uab.cat/web/organ-habilitat/composicio-1345736961519.html; Project Ref: CEEAH-1226) and adhere to the Spanish National and Institutional guidelines and regulations (Directive 2010/63/UE)

### Selection by animal personality

Fish from G1 were separated by their behavioural response in a group risk-taking test. In previous studies this test was validated as a good screening method for proactive and reactive zebrafish [13], highly correlated to other personality tests performed previously (mirror stimulation test, feeding response, activity and novel environment) and animals were consistent in their responses over time. The risk-taking test in group measures the time an individual take to leave the group from the safety of a shelter. It highlights an individual’s predisposition to explore a new and potentially dangerous environment. The behavioural tank, 20 L (40 x 20 x 25 cm), had opposite and lateral sides of the observation arena covered to reduce stress and isolate from the outside world (see S1 Fig). The tank was divided using a black Polyvinyl chloride (PVC) sheet used as a separator, creating a small sheltered area completely covered (1/3 of the total area of the tank). The separator included a centred hole of 3 cm diameter, located at 10 cm from the tank bottom. The new environment consisted of the remaining open space in the tank. A group of nine fish were transferred from the holding tank and placed into the sheltered area. The hole of the separator was covered with a black, sliding sheet of the same material. Fish were acclimatized for 10 minutes to reduce the handling stress and acclimate. The test was divided in 2 phases of observation. When the 10-minute acclimation period ended, the sheet that covered the hole was carefully removed, allowing the fish to decide to stay in the shelter or exit into the new environment. This first phase of observation lasted ten minutes. The first three fish that entered the new environment, or the fish with a latency of lower than 10 minutes were selected as P, recording the total latency to exit for each individual fish. After 10 minutes the exit hole was closed, and the P individuals moved to a new stock tank labelled (P). Time was continuously recorded. A second period of observation of 15 minutes was then initiated by removing the hole cover. The first three fish that entered the new environment or if less than three entered in 15 minutes were selected recording the total exit latency for each individual.

The exit hole was sealed once again, and these individuals were discarded for the purpose of the experiment. All remaining fish in the shelter were classified as R and placed in a separate holding tank (R).

### Female guarding behaviour

A sub-sample of 8 males selected per behavioural phenotype (P-R personalities) were placed individually into a 38-40L (50x50x25cm) tank with a small breeding tank in the centre (15x10cm; with marbles at the bottom of the tank). Individual males were left for 12h (overnight) into the experimental breeding tank. The next morning, we placed an intermediate mature female in the tank and observed the behaviour of the male regarding the female for 15min. An oval around the breeding tank was drawn with a permanent marker (10cm from the sides and 15 cm from the front and back of the small breeding tank) as the observation area. Females were left 30 min to acclimatise before being recorded (in sec) for 15 min. we recorded the time the male kept the female within the circle and close to the small breeding tank as a measure of female guarding behaviour and territoriality.

### Experimental design

This experiment was designed to evaluate the effect of personality on the reproductive success and larval survival in *D*. *rerio*, as a measure of fitness on two colour phenotypes (WT-UAB and WT-I for G1 generation). We had both colour phenotypes within P and R individuals. A total of 10 P and 9 R G1 pairs, coming from the selection program, were crossed for both colour phenotypes. Six of the Proactive and five of the Reactive pairs were WT-UAB, the rest (4 pairs each) were WT-I phenotype (Fig 2). Two weeks before starting the experiment fish were weighed and the standard and total length was recorded. The Fulton condition factor for each of the experimental subjects was determined [57,58]. Each pair performed three successful spawning events during the test. The eggs were extracted and set on a 140 mm diameter Petri dish. A total count of the eggs was recorded to determine if it was a *successful spawning*. A spawning was considered successful if more than 30 fertilized embryos were observed during the count. Spawning with less than 30 fertilized embryos were discarded following the criteria that a minimum number of embryos were needed to get acceptable numbers of fish at 30dpf considering the percentage of mortality. Every successful spawning was incubated with E3 medium [59] in 55 mm diameter Petri dishes as a common laboratory procedure. At the end of the embryonic stage, the survival of each of the families was evaluated. The larval stage of the G2 progeny was also evaluated to assess the personality effect of the parents in the offspring performance. We recorded larval survival and growth (total length) for each of the G2 families.

#### Collecting, cleaning and management of spawning

After transferring the brood stock pairs from each family spawning tank to their respective stock tanks, floating farrowing spawn was removed and examined for the presence of eggs. The eggs were extracted by pouring E3 medium with the aid of a squeeze bottle and were collected in a fine mesh aquarium, carrying out three consecutive washes using E3 medium. Subsequently, the eggs were transferred into a Petri dish of 140 mm diameter. With the aid of a 3mL Pasteur pipette, any remaining food or stool was removed. The total number of eggs laid was counted. Eggs with some opaque colouring or malformations were discarded. The clean eggs were transferred to a Petri dish of 95 mm containing 11mL of E3 medium, labelled and left for 24 hours to check the embryonic viability. Next morning infertile eggs and dead embryos were removed (1dpf viability). Viable embryos from each family were placed again in groups of 30 in a 55mm diameter Petri dish in 11 ml E3 medium and labelled with their origin family data. Temperature was always maintained at 28°C, light-dark cycle was at 14:10 hours.

#### Spawning

A spawning was considered successful if more than 30 fertilized eggs [60,61], 2–4 hours after sunrise, were observed. The *Spawning variables* analysed were: the *spawning rate* (SR) defined as the number of successful spawning divided by the total number of crossing pairs within each behavioural category multiplied by 100 [61]. This gives an indication of the fertility (related to embryonic viability) or number of offspring born per mating pair. *Total eggs spawned* or *Total spawning* per pairs include viable fertilised eggs, unfertilised eggs, opaque colouring eggs and malformed embryos that died during the first 24 hours, per pairs. This is considered total fecundity or potential for reproduction [62,63]. *Successful spawning* per pairs, defined as the number of viable embryos produced at each spawn and then divided by the number of pairs. *Spawning viability* per pairs calculated as the number of viable embryos 24 hours’ post-fertilization, divided by the total number of eggs spawned for all the successful spawns, multiplied by 100. Both are also measurements of fertility and egg viability per crossing pairs.

#### Embryonic stage

The *successful spawning* of each pair was separately incubated in 55 mm diameter Petri dishes. Thirty embryos were placed in each Petri dish containing 11 ml of E3 medium (density: 2.72 embryos/ml). Daily monitoring of the temperature of the room was performed (28 ±1°C). The light dark cycle was maintained at 14:10 hours. Embryos hatched between 2 to 3 dpf. The chorion was removed with the aid of a Pasteur pipette 3ml. Completed 72 hpf, embryo viability was recorded [64]. Live larvae were again transferred to Petri dishes of 55 mm, containing 11 mL of fresh E3 medium.

#### Larval stage

After the embryonic stage and at the beginning of the larval stage (3 dpf), three larvae of each breeding pair were selected at random and full length was registered, then they were returned to their respective Petri dishes. This was recorded periodically at 7, 14, 21 and 30 dpf to follow ontogenetic development stages. Larvae at 5dpf were transferred and randomly distributed into 2.8 L methacrylate tanks (Aquaneering**®** ZT280; Dimensions (L X W X H): 26.5 X 10 X 14 cm) by adjusting the density and starting the following feeding recommendations established by the food supplier (ZfBiolabs®). Control of daily mortality was performed until the end of the experiment, at 30 dpf (when at a juvenile stage). At the end of the larval stage, *larval growth* and *viability of the offspring* (juveniles) for each of the breeding pairs was recorded. *Larval growth* was determined using the length (mm) from the tip of the upper jaw to the upper tip of the caudal fin. *Larval viability* was defined as those fish larvae with active movement 30 days after fertilization.

### Statistical analysis

All data were tested for normality using Shapiro-Wilk´s and Levene's tests for the homogeneity of variances. Data on female guarding was analysed by a two-tailed non-paired samples T-test. Some non-normal fitness related data was being normalised by applying square root transformation (larval viability) and the morphometric and other fitness non-normal data was analysed with a Kruskal-Wallis non-parametric test (weight, condition factor (K), total spawning, spawning rate and larval growth).

Normal data was analysed by Two-way ANOVA test to determine differences between colour and behavioural phenotypes for total length and some fitness related variables (successful spawning events per pairs, percentage of spawning viability at 1dpf and embryonic viability at 3dpf), followed by LSD *post hoc* comparisons. All pairwise comparisons extracted from the LSD *post hoc* matrix were corrected for multiple comparisons using the sequential Bonferroni correction.

Overall survival of brood, from egg to larvae, from different behavioural and colour phenotypes pairs was assessed using the Kaplan-Meier non-parametric survival estimator.

All statistical analyses were performed on the statistical software packages STATISTICA version 7 (StatSoftV7**®)** and Graph Pad Prism V.6 (San Diego, CA, USA).

## Results

### Behavioural data

For G1 we could form 10 pairs (n = 20 fish) with a proactive personality and 9 pairs (n = 18 fish) with a reactive personality. From the 10 pairs with a proactive personality, 6 (n = 12 fish) had a homogeneous colouration pattern and the rest had heterogeneous colouration patterns. From the 9 reactive pairs, 5 (n = 10 fish) had a homogeneous colouration and 4 (n = 8 fish) were heterogeneous (see Fig 2).

For the female guarding behaviour we found proactive males (mean±SD: 464±81.2) kept the females closer to the reproductive area for longer than reactive males (mean±SD: 376± 80.37) and it was significantly different (T-test; t(14) = 2.16; p<0.05). Both P and R males displayed guarding behaviours against the females to keep them close to the small breeding tank, but we did not quantify the different behaviours or the intensity of this behaviours.

### Morphometric data

No significant differences in weight (K-W_3_ = 0.49, p = 0.91), total length (two-way ANOVA: for personality F_1,34_ = 0.25, p = 0.62, colour F_1,34_ = 2.53, p = 0.12 or their interaction F _1, 34_ = 4.01, p = 0.05) or Fulton condition factor (K) (K-W_3_ = 2.39, p = 0.49) among colour or behavioural phenotype P and R breeding families were found. The only differences were found in the ocular orbital diameter between Proactive WT-I (mean±SD = 2.15±0.07) and Reactive WT-I (mean±SD = 2.09±0.06, p = 0.025; N = 40)

### Reproductive stage

A total of 128 crosses, where 57 successful spawns were achieved, produced 13,664 eggs, of these, 10,362 were viable eggs (2–4 hpf) followed during the subsequent phases of the study (see in more detail for each colour and behavioural phenotype in Table 2).

**Table 2:** Reproductive behaviour of zebrafish P and R lines for the first generation G1 in both colour phenotypes.

**Phenotype**	**Personality**	**Number of Crossings**	**Number of Successful Spawning**	**Total Number of Eggs**	**Number of Viable Eggs 2-4hpf**	**Percentage of Spawning (%)**
**WT-UAB**	P	39	18	4 528	3 704	**46,43%**
	R	30	15	3 828	2 921	**50.57%**
**WT-I**	P	31	12	3 054	2 234	**38.84%**
	R	28	12	2 254	1 503	**43.30%**
**Total**		**128**	**57**	**13 664**	**10 362**	

### Spawning and Viability

#### Total Spawning

**Fig 3:**
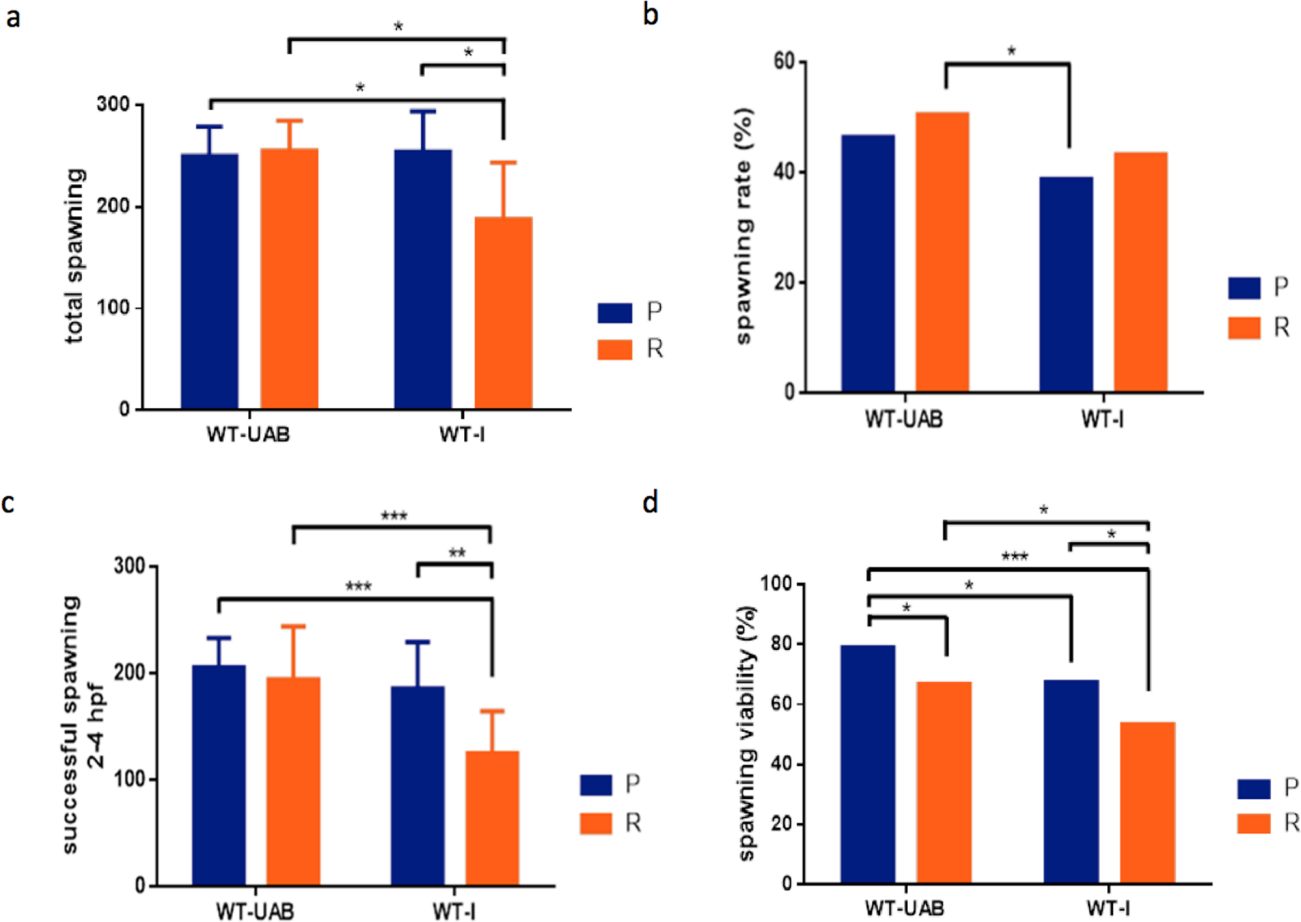
Reproductive variables measured from generation 1 (G1) crossing pairs. (a) **Mean** (± SD) total number of eggs (spawning) per personality and colour phenotype per pairs. (b) Spawning rates (in percentages) for different personalities and colour phenotypes. (c) Mean (± SD) average size of successful spawns per pairs (more than 30 fertilized eggs viable) by personality within colour phenotype (WT-UAB and WT-I) for the first generation G1. (d) spawning viability in %. The data represents the average of three spawns for each of the pairs and two phenotypes. *p <0.05, **p<0.01 and *** p < 0.001.

All comparisons between personalities and colour phenotypes and their interaction resulted in significant differences (K-W_3_ = 12.41; p = 0.006). Significant differences in spawning were not due to differences within the homogeneous colour phenotype (WT-UAB) between personality types but due to significant differences between colour phenotypes and between personalities in the WT-I phenotype (see Fig 3A for specific statistical significances).

#### Spawning rate

No significant differences between P and R, in spawning rate, were found (U = 29.5, p = 0.21). We didn’t detect any significant interaction between parent personality and colour phenotype (K-W_3_ = 9.58; p = 0.02). However, we detected significant differences between colour phenotype in spawning rate (U = 13, p = 0.005). Differences observed were between the Reactive WT-UAB with the higher values and the Proactive WT-I with the lower spawning rate (50.57% and 38.84% respectively). These data identify that lower spawning rates are within the P groups of heterogeneous colouration phenotypes (P WT-I, see Fig 3B).

#### Successful spawning events per pairs

Significant differences between P and R, in successful spawning events per pairs were found (2-way ANOVA: F _1, 53_ = 11.01, p = 0.001). We detected significant differences between colour phenotype and successful spawning (2-way ANOVA: F _1, 53_ = 16.88, p< 0.001). Similarly, we detected a significant interaction between personality and colour phenotype (2-way ANOVA: F _1, 53_ = 5.29, p = 0.02) for this particular case (see Fig 3C).

#### Percentage of spawning viability at 1dpf

Fish with personality P had a higher percentage of the fish reproductive viability (2-way ANOVA: F _1, 53_ = 19.95, p<0.001). We detected significant differences between colour phenotypes in percentage of spawning viability (2-way ANOVA: F _1, 53_ = 18.37, p< 0.001). However, we did not detect any significant interaction between parent personality and colour phenotype (2-way ANOVA: F _1, 53_ = 0.10, p = 0.75). Differences were between the Proactive WT-UAB with the higher, Reactive WT-UAB, Proactive WT-I and the Reactive WT-I with the lower spawning rate (79.05%, 66.93%, 67.46% and 53.47% respectively, see Fig 3D).

### Embryonic stage and viability

#### Embryonic viability at 3dpf

Dead and deformed embryos were removed every day until the end of the embryonic stage at 3 dpf. Total numbers of embryos hatched were compared with the initial numbers of embryos at 1dpf. *Embryo viability* was over 90% for both types of personalities overall and for both colour phenotypes.

No significant differences between embryos coming from P and R parents in fish embryo viability was found at 3dpf (2-way ANOVA: F _1, 53_ = 0.08, p = 0.77). No significant differences between colour phenotype and embryo viability (2-way ANOVA: F _1, 53_ = 1.03, p = 0.31) or parent personality and colour phenotype (2-way ANOVA: F _1, 53_ = 0.29, p = 0.59) were detected at this stage.

### Larval stage and viability

#### Larval viability and growth

Significant differences between P and R parents in larval viability were found (2-way ANOVA: F _1, 53_ = 8.14, p = 0.006). We did not detect any significant differences between colour phenotype and larval viability (2-way ANOVA: F _1, 53_ = 0.36, p = 0.54) and no significant interaction between personality and colour phenotype in larval viability were observed (2-way ANOVA: F _1, 53_ = 0.67, p = 0.41).

**Fig 4:**
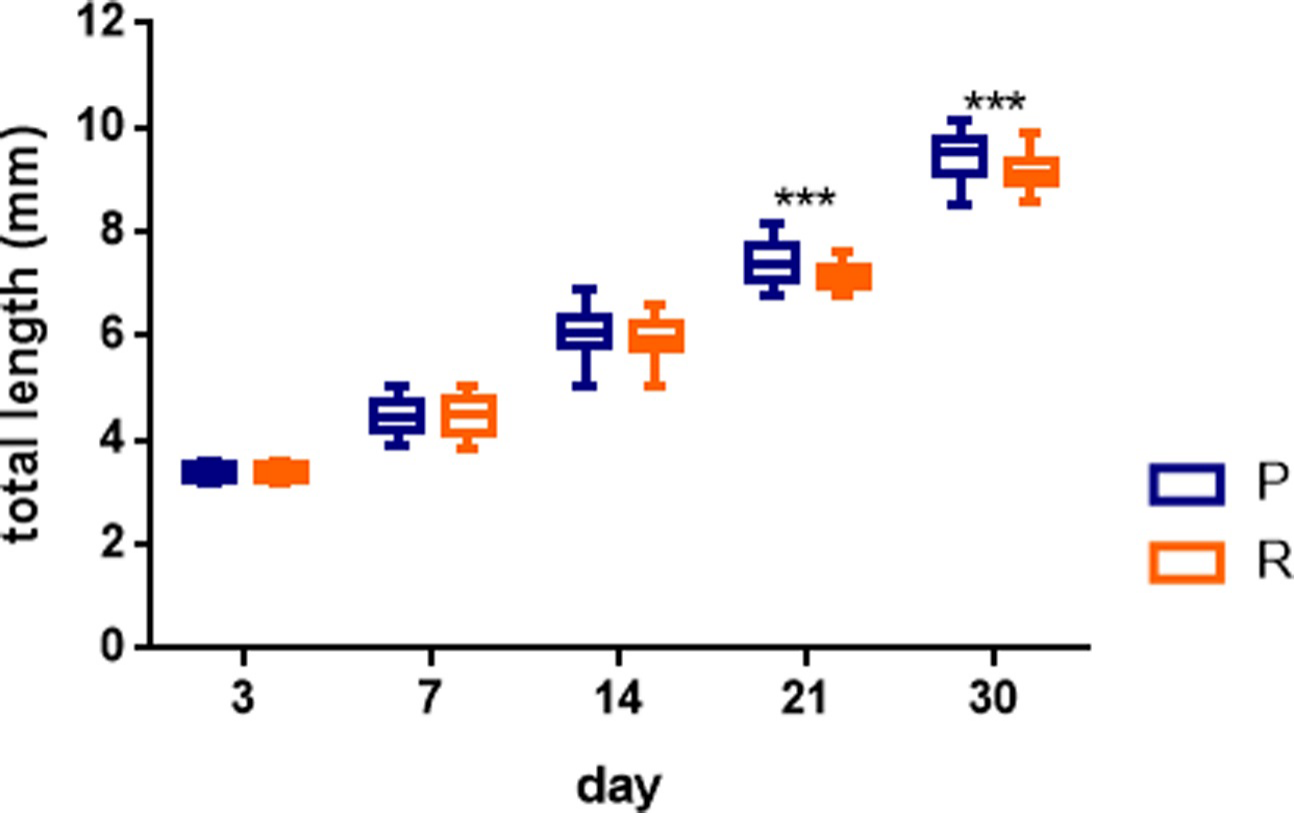
Larval growth. Total length (in mm) from the beginning to the end of the larval stage between families’ P and R (***p<0.001). Differences were significant from 21 dpf until juveniles (30dpf). Individuals from P families had a greater overall growth than R individuals for both colour phenotypes.

Larvae from personality P parents have a faster growth rate than larvae from R parents after 21dpf (K-W_3_ = 37.75, p<0.001; see Fig 4). At 30 dpf there were also differences between P and R within the same WT-UAB and WT-I colour phenotypes and between P groups with different colour phenotypes (K-W_3_ = 37.72, p<0.001; see S2–S4 Figs for detailed significant differences between personalities and colour phenotypes at 21 and 30 dpf).

#### Total survival

**Fig 5:**
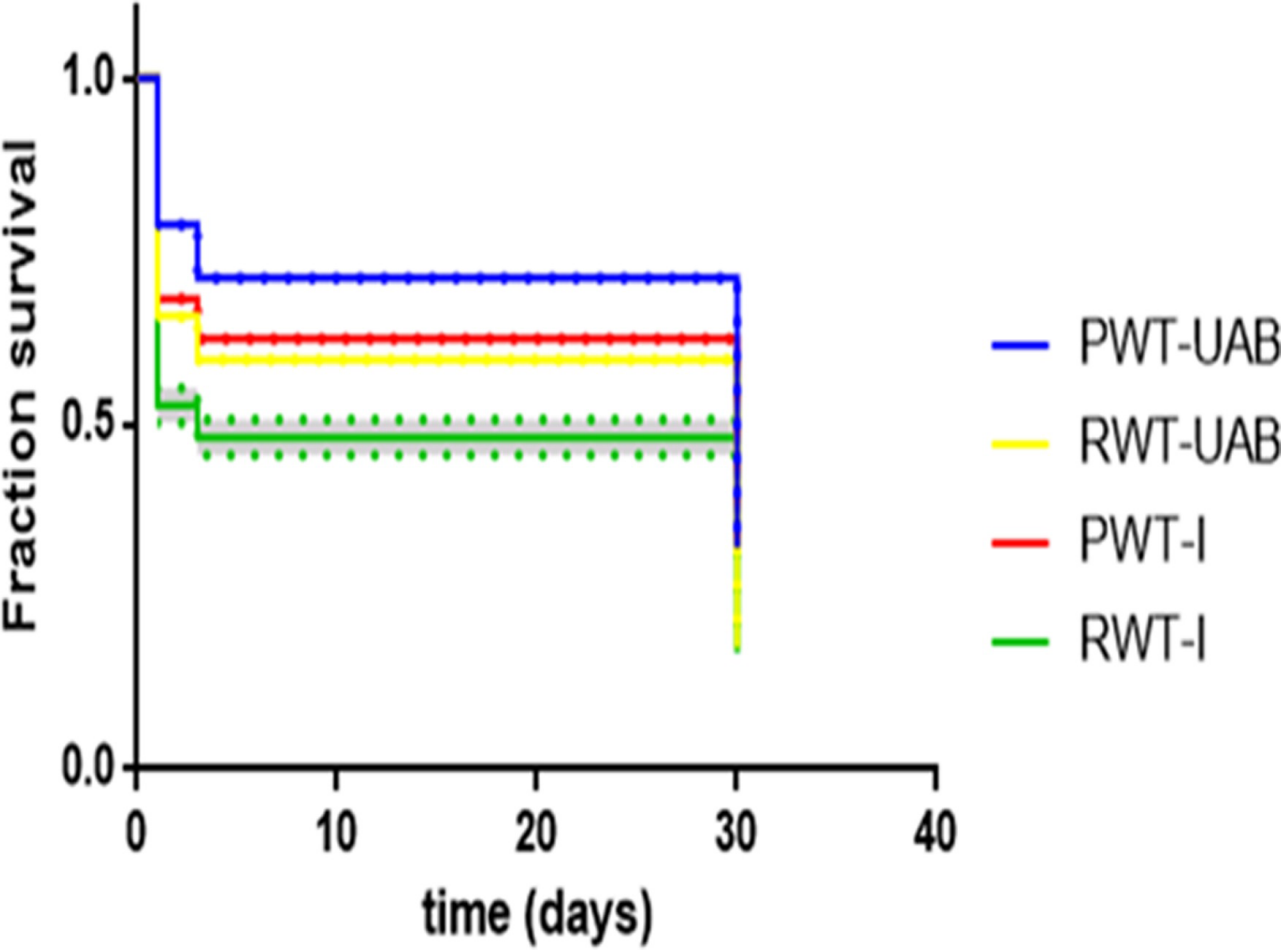
Total individual survival. Total survival graph where individuals from P families for both colour phenotypes show a better overall survival at all developmental stages.

P individuals of both colour phenotypes had a higher percentage survival than R individuals (P WT-UAB = 32.05%; P WT-I = 31.20%; R WT-UAB = 17.66%; R WT-I = 16.90%, respectively). The Kaplan-Meier test shows a strong interaction between personality and colour phenotype in survival (χ^2^ (3) = 359.1, p< 0.001), see Fig 5.

## Discussion

This study brings new insight into the reproductive success of males and females from a breeding program of zebrafish (*Danio rerio*) where selection for morpho-type, colour phenotype and behavioural phenotype, was combined under controlled constant laboratory conditions. Our results show that the Proactive behavioural phenotype in zebrafish has greater reproductive success than their Reactive counterparts across all morpho-colour phenotypes. However, comparisons between both colour phenotypes shows that the homogeneous WT-UAB phenotype had better reproductive rates than the indefinite WT-I phenotype in general and within the personality phenotypes. Furthermore offspring (larval stage) from P fish had a higher percentage of survival and higher growth rate than offspring of the R line. The performance between both colour phenotypes was similar for embryonic and larval stages meaning that parental colouration seems only to have an effect at initial reproductive stages.

### Reproductive success

The main result of our study is that there are differences in reproductive success between P and R individuals previously screened for personality with a risk taking in group test [24,65]. The P fish show greater viability of spawning than R fish, that was independent of environmental conditions as fish were assessed under the same constant laboratory conditions. This result has important consequences for offspring survival. As zebrafish do not have parental care, a greater number of viable spawning events increase the probability of a greater number of offspring that will have a greater chance of survival and thus more fitness for the species [66].

Across our experimentation, and as a general trend, bold individuals a characteristic behaviour of individuals of the P personality, have greater reproductive success than shy individuals of R personality [67]. Similar results have been reported in bighorn sheep [35], black rhinoceros [68] and rainbow fish males extrapolating from their position in the hierarchy rank [69]. For fish some studies combining behavioural traits related to mating and external colouration also found differences in guppies reproductive success, with more dominant males performing a more intense courtship being more successful with a different relative importance of colour [3]. In zebrafish, Ariyomo and Watt reported that bold and aggressive males have a higher spawning viability than shy individuals [36]. Our results corroborate this observation where P individuals showed greater spawning viability than individuals R. However, our results revealed no difference in weight, total length and condition factor between P and R in contrast to that reported for other species like natural populations of the poeciliid *Brachyrhaphis episcopi* [70] where bold animals showed higher body mass. In our experimental set-up this was not found, it could mainly due to lower numbers but also could be an effect of domestication as our fish are under laboratory rearing conditions with no food restriction whereas the poecilid study was done with natural populations. Although in our study where proactive fish were more successfully reproducing than reactive fish, there were no correlations to size differences. Size differences can be related to different metabolic rates, as reported in literature [15,26,54] but this wasn’t tested in our experiment. Therefore, personality may play an important role in reproductive success in zebrafish.

Increased viability of spawning may be related to a better synchronization between males and females during the liberation of gametes. According to Harvey et al.[71], the zebrafish sperm once free in the water had mobility around 60 seconds that is approximately the same time the egg becomes activated once in the water [72]. This may be the result of the behaviour adopted by P individuals, observed in our experimental setup, where the P male displays a more aggressive mating behaviour and as a result they keep the females close to the reproductive area for longer. This is characterised by intense swimming around the spawning site, close bodily contact to capture olfactory cues that initiate ovulation [73] therefore ensuring fertilization of the greatest number of eggs at the time of spawning [74]. On the contrary, R individuals have a less aggressive behavioural pattern, not only restricted to the spawning site, chasing the female through the laying tank until spawning [52]. Reactive fish had more spawning events of fewer quality eggs. That could give them an advantage in high predator pressure habitats: reactive personalities tend to hide and avoid predators better than proactive [30]. Over an extended period of time this might be a more successful reproductive strategy.

Another possible reason for the viability of spawning may be determined by the male sperm quality however reports are contradictory and could not determine a relationship between personality and sperm quality [31,75].

The results for the other reproductive variables studied: rate of spawning and the number of healthy embryos for every successful spawning, showed no significant differences. These results are similar to those found by Castranova et al. in [60], that found no significant differences between the average sizes of spawning and spawning rate in eight different laboratories, but the authors highlighted that in some laboratories the density has an effect on the percentage viability where lower densities are related to lower viability. Based on this background, a possible explanation for the lack of difference in spawning rate may be the fact that there is no competition between the females for the spawning site where the dominant female suppresses the spawning of subordinate females through hormonal mechanisms [66,76]. For males, a possible explanation for similar values of spawning rates and the number of embryos is that mating type is done in pairs where the male does not have to engage with aggressive behaviour toward other males that interferes with the mating process [33,77].

The homogeneous colour phenotype, WT-UAB showed a consistent higher reproductive success independent of personality. Individuals P and R colour phenotype WT-UAB, showed a higher reproductive success than that shown by individuals P and R colour phenotype WT-I. A possible explanation for the difference is the greater uniformity in the WT-UAB phenotype resulting from this selection whereas WT-I always presents higher variability in the population.

### Survival and growth

Our results show that P larvae have longer survival and higher growth than R larvae at the end of the larval stage. This seems to be directly related to the reproductive success of the parents, suggesting that personality can have a heritable component [78–81]. Assuming there is a heritable component of personality, longer survival and higher growth in the offspring of parents’ P may be related to a greater willingness to take risks and explore the new environment and thus achieve greater access to resources [6,82,83].

At the level of natural populations, a possible implication of faster growth is to achieve sexual maturation at an earlier age. However, reproductive strategies exhibited by both behaviours may be more difficult to interpret and may be influenced by various exogenous factors such as predation [78,84], or resource availability [85,86] that can have an impact on the reproductive dynamics exhibited by an individual. For example, a P individual with a higher risk-taking behaviour is likely to have a higher percentage of predation therefore reproductive success has to be higher than a R individual with lower reproductive success but a longer reproductive life due to lower levels of predation by taking lower risks [15,67]. If the natural reproductive behaviour of this species is taken into account where it is associated with the monsoon season, then the availability of food is much higher. During this period offspring should achieve better body condition to ensure survival in times of food scarcity, and the appropriate conditions for reproduction in the following season. However, this is just speculative as there are no direct observations describing predation risk of natural populations of zebrafish in the current literature to sustain this claim. Field studies show the presence of piscivorous fish living in the same habitat that could be potential zebrafish predators [40,41]. Indirect experiments on predation under laboratory conditions show zebrafish displaying fright reactions in response to potential predators in the wild [87].

In conclusion, this study demonstrates that proactive zebrafish have greater reproductive success than reactive individuals under constant laboratory conditions. This is highlighted by increased viability and better growth performance in P offspring. Our results show the combined effect of personality traits and colouration patterns on reproductive success. These results have important implications for the fitness of the species in the wild and when bred in captivity. In the wild the interaction between colouration patterns and personalities could be a driver of speciation by differential mating success and assortative mating, differential mortalities at earlier developmental stages and differential growth rates. They are also important for other commercial fish species breed under common aquaculture practices where selection for external appearance or behavioural traits might also select for correlated traits, some of which could have significant economic implications such as increased aggression or better survival and growth rates at larval stages.

## Supporting information

S1 Fig Experimental set-up of the risk-taking tank for the behavioural screening. The tank has a sheltered area (1/3 of the tank) with all sides and top covered. The open area covers 2/3 of the tank and represents a novel environment. Both sides are separated by a PVC sheet with a whole in the middle to allow the fish to cross between areas.
(TIF)

S2 Fig Larval growth. Significant differences between colour phenotype and larval growth (p<0.001) were also observed for 21dpf larvae and juveniles (at 30dpf). *p <0.05 and **p<0.01.
(TIF)

S3 Fig Larval growth at 21 dpf. Detailed significant differences in total length between personalities and colour phenotypes from 21 dpf larvae. R WT-UAB vs P WT-UAB ***p<0.001; P WT-I vs P WT-UAB **p = 0.002; R WT-I vs P WT-UAB ***p<0.001.
(TIF)

S4 Fig Juvenile growth at 30 dpf. Detailed significant differences in total length between personalities and colour phenotypes from 30 dpf juveniles. R WT-UAB vs P WT-UAB ***p<0.001; P WT-I vs P WT-UAB *p = 0.02; R WT-I vs P WT-UAB ***p<0.001.
(TIF)
