# Supplementary figures and images for: ‘Love at first sight’: The effect of personality and colouration patterns in the reproductive success of zebrafish (Danio rerio)
Source: PLoS One. 2018 Sep 19;13(9):e0203320. doi: 10.1371/journal.pone.0203320 (PMC6145548; doi:10.1371/journal.pone.0203320)

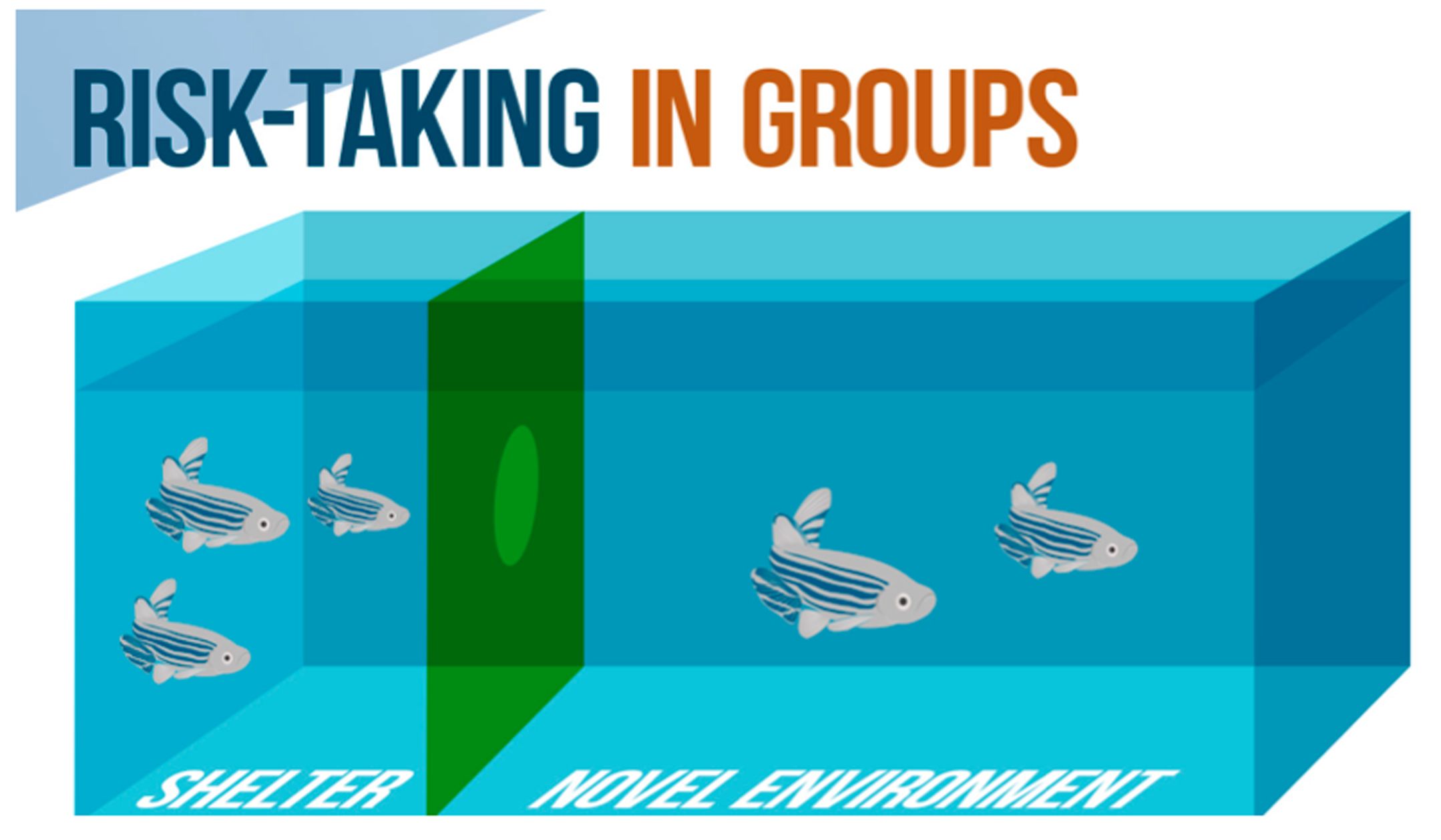

Supplement: S1 Fig — The tank has a sheltered area (1/3 of the tank) with all sides and top covered. The open area covers 2/3 of the tank and represents a novel environment. Both sides are separated by a PVC sheet with a whole in the middle to allow the fish to cross between areas. (TIF) [file pone.0203320.s001.tif]

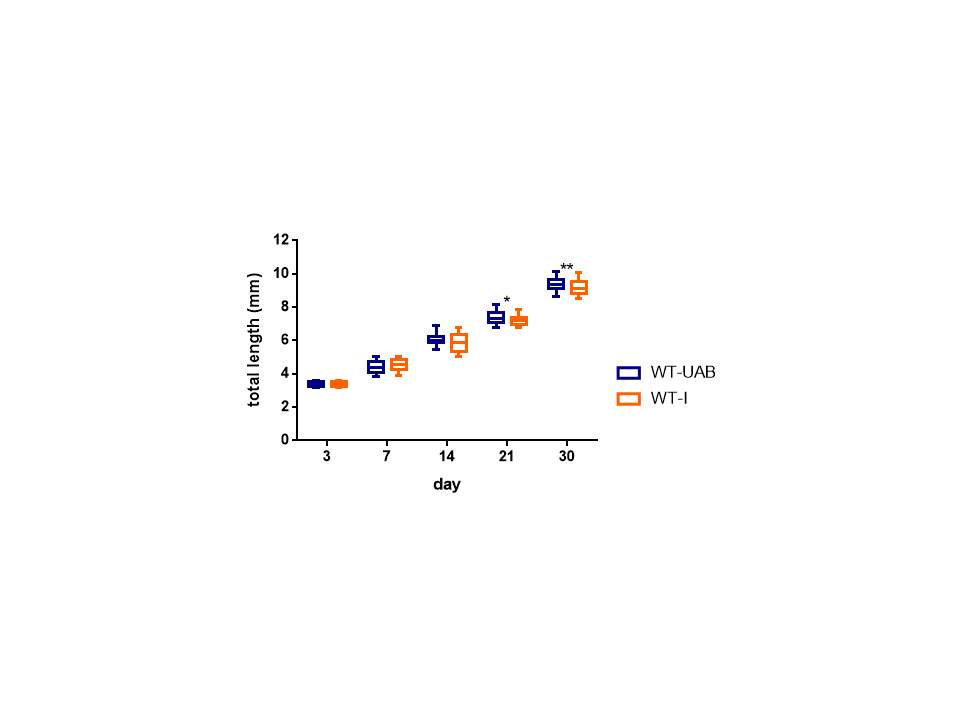

Supplement: S2 Fig — Significant differences between colour phenotype and larval growth (p<0.001) were also observed for 21dpf larvae and juveniles (at 30dpf). *p <0.05 and **p<0.01. (TIF) [file pone.0203320.s002.TIF]

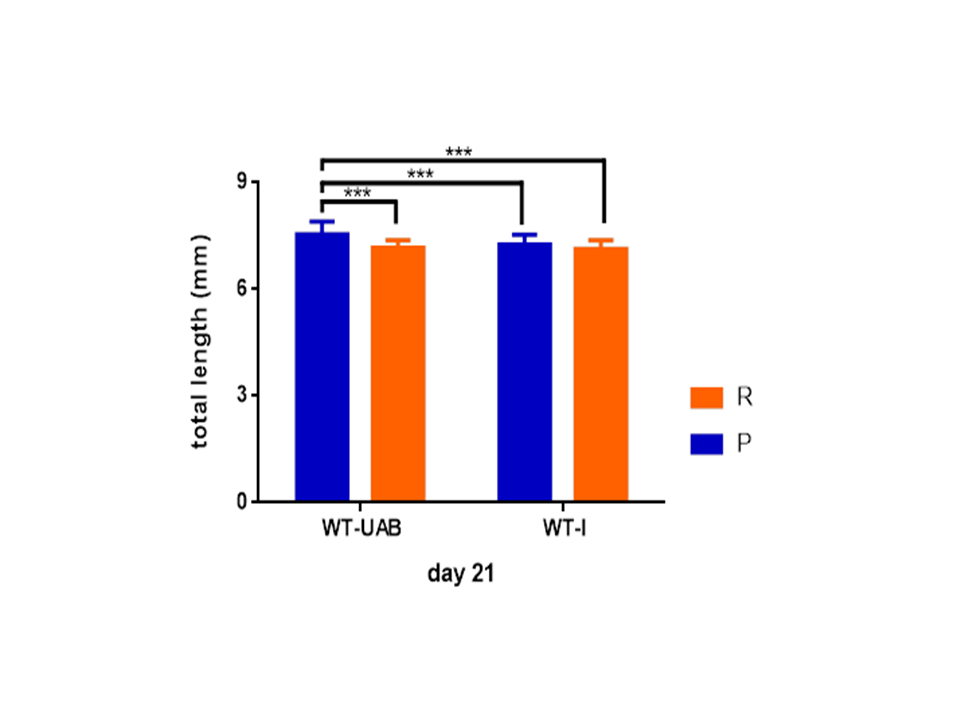

Supplement: S3 Fig — Detailed significant differences in total length between personalities and colour phenotypes from 21 dpf larvae. R WT-UAB vs P WT-UAB ***p<0.001; P WT-I vs P WT-UAB **p = 0.002; R WT-I vs P WT-UAB ***p<0.001. (TIF) [file pone.0203320.s003.tif]

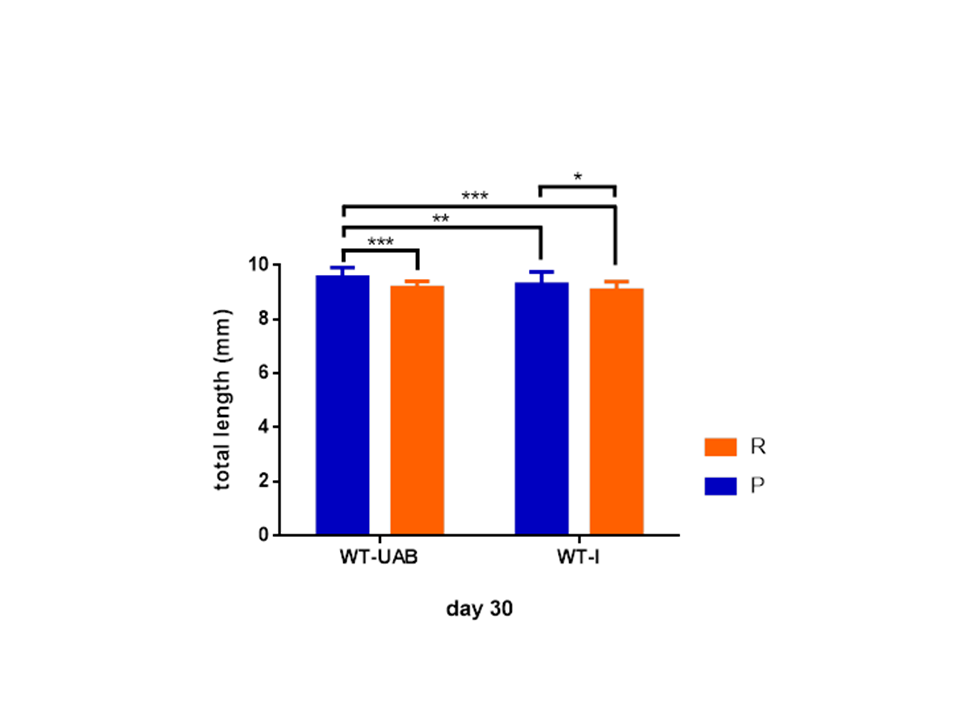

Supplement: S4 Fig — Detailed significant differences in total length between personalities and colour phenotypes from 30 dpf juveniles. R WT-UAB vs P WT-UAB ***p<0.001; P WT-I vs P WT-UAB *p = 0.02; R WT-I vs P WT-UAB ***p<0.001. (TIF) [file pone.0203320.s004.tif]
